# Accuracy of thoracic pedicle screw placement in adolescent idiopathic scoliosis patients using the entry point identified by new landmarks: a computed tomography study

**DOI:** 10.1186/s12893-022-01827-1

**Published:** 2022-11-04

**Authors:** Jun Jiang, Xu Chen, Yong Qiu, Bin Wang, Ze-zhang Zhu, Yang Yu

**Affiliations:** 1 Division of Spine Surgery, Department of Orthopedic Surgery, Nanjing Drum Tower Hospital, The Affiliated Hospital of Nanjing University Medical School, Zhongshan Road 321, Nanjing, 210008 China; 2 Medical School of Nanjing University, Nanjing, China

**Keywords:** Thoracic, Pedicle screw, Adolescent, Accuracy, Scoliosis

## Abstract

**Background:**

Although thoracic pedicle (TP) screw has gained increasingly popularity in the surgical treatment of adolescent idiopathic scoliosis (AIS) patients, questions remain about the accurate selection of entry point for TP screw placement in these patient. The main objective of the present study was to evaluate the accuracy of TP screw placement in AIS patients using the entry point identified by new landmarks.

**Methods:**

Thirty-four thoracic AIS patients treated with posterior TP screw instrumentation were included. All these TP screws were inserted through the entry point identified by new landmarks with free-hand technique. Postoperative CT scans were obtained to evaluate the screw position. The perforations of the pedicle were classified as grade 0 (no perforation), grade 1 (≤ 2 mm), grade 2 (2.1–4 mm), grade 3 (4.1–6 mm) and grade 4 (6.1–8.0 mm). Screws in grade 0, displaced either medially or anteriorly in grade 1 and displaced laterally in grades 1 to 2 were considered acceptable.

**Results:**

Of the 495 TP screws inserted, 34 (6.9%) screws were displaced with 7 screws (1.4%) displaced medially, 20 screws (4.1%) displaced laterally and 7 screws (1.4%) displaced anteriorly (P < 0.05). Among the 34 displaced screws, 11 screws (32.4%) were considered as grade 1, 14 screws (38.2%) as grade 2 and 9 screws (29.4%) as grade 3 (P < 0.05). The overall rate of acceptable screws was 97.8%. No screw-related complication was noted.

**Conclusion:**

Our new method for selecting the entry point of TP screw in AIS patients is convenient and can achieve high accuracy of screw placement, which is worthy of being widely popularized.

## Background

Nowadays, the application of thoracic pedicle (TP) screw has gained increasingly popularization in the surgical treatments of adolescent idiopathic scoliosis (AIS) patients due to its advantages such as better correction rate, shorter instrumentation length and less loss of correction as compared to the conventional internal fixations [1–3]. However, TP screw insertion is technically demanding in scoliosis patients due to both the changed anatomy of thoracic pedicle and the presence of vital organs adjacent to the thoracic vertebra, with perforation rates ranging from 1.2 to 65.0% [4–9].

To improve the accuracy of TP screw placement, various screw placement methods, such as free hand technique, funnel technique, fluoroscopy and computer-assisted surgery had been described [10–13]. However, accurate TP screw placement depended on an accurate entry point no matter which method was taken since the margin of error for TP screw was very low. Several scholars had described different entry points of TP screws at different levels to guide the screw insertion [14–17]. However, most of these guidelines were complex and some had been proven to be inappropriate due to the high incidences of screw malposition, which caused confusions on selecting the ideal entry points, especially for the beginners without much experience on screw placements.

To facilitate the TP screws placement, we proposed an ideal pedicle entry point which can be easily identified by new landmarks. In this current study, we evaluate the accuracy of TP screws placements in AIS patients using this entry point with free-hand technique to verify the effectiveness and superiority of this novel method.

## Materials and methods

### Subjects

A total of 34 consecutive thoracic AIS patients underwent posterior correction surgery using TP screws in our institution from August 2018 to June 2019 were included. There were 25 female and 9 male patients with a mean age of 15.0 years at the time of surgical treatment. There were 30 Lenke type 1 curves and 4 Lenke type 2 curves with the average main thoracic curve of 50.7°. All the TP screws were inserted by the first author (J.J.) using a new method of selecting entry point. All these patients had postoperative CT scans of the thoracic spine. This study was approved by the institutional review board in our hospital.

### Surgical techniques

**Fig. 1:**
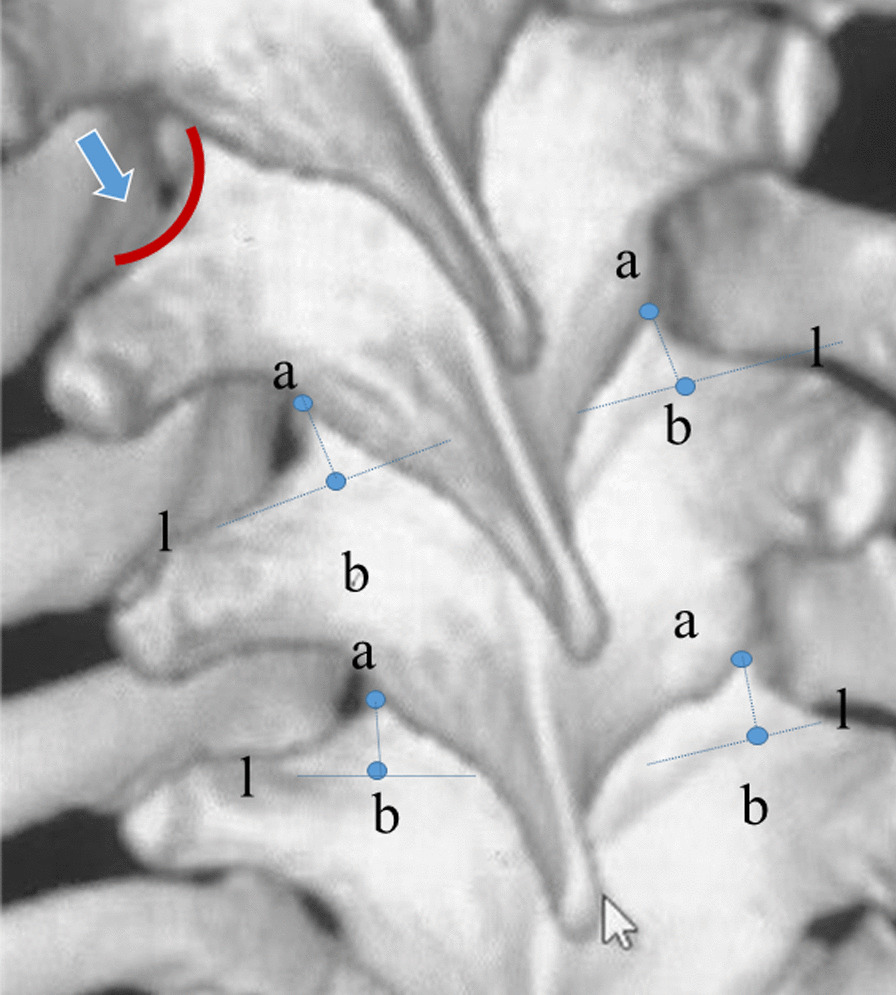
Illustration of selecting entry point for TP screws. Red curve (arrow): the curved border connecting lateral border of the superior facet and the transverse process. Point a: the intersection of the curved border and inferior facet; line l: the base of superior facet; point b (entry point): the junction of line l and the line through point a, paralleling to the supraspinous ligaments

After a standard midline incision, the posterior parts of the thoracic spine were exposed with sub-periosteal dissection laterally to the transverse process. The facet joints were meticulously cleaned of soft tissue to show the bony landmarks better. After cleaning the soft tissue, a curved border connecting lateral border of the superior facet and the transverse process can be found. The entry point was at the junction of the base of superior facet and the line through the intersection of the curved border and inferior facet, paralleling to the supraspinous ligaments (Fig. 1). After identifying the entry point, a pilot hole of 3–5 mm indicating the pedicle entrance was created with an awl. A pathfinder was advanced into the pedicle with a slight ventral pressure. After inserting approximately 15–20 mm, the pathfinder was removed and a pedicle sound was used to feel the intact medial, lateral, superior, inferior cortices of the pedicle. If any perforation of the cortex was detected, the direction of the pathfinder was changed. Subsequently, the pathfinder was further advanced into the vertebral body until its tip met the anterior cortex. Then the pedicle sound was used again to finally check the entire length of pedicle screw trajectory. The diameter of the TP screw was 4.5–5.5 mm in upper thoracic spine (T1–T4), 5.5 mm in middle thoracic spine (T5–T8) and 5.5–6.5 mm in lower thoracic spine (T9–T12). The length of the TP screw was determined by measuring the maximal inserted depth of the pedicle sound. After finishing the procedure of TP screw placement, resection of inferior articular process was performed in each thoracic vertebrae fused and the curve was corrected by translation technique. The neurophysiological monitoring were continuously performed during the operation.

### CT measurement

Postoperative CT scan was performed in all the patients. The screw perforations were evaluated on PACs image system (version 5.0, GE Healthcare). The perforations of the bony cortex were measured in millimeters and were divided into five grades: grade 0 (fully contained within the pedicle), grade 1 (perforation ≤ 2 mm), grade 2 (perforation 2.1–4.0 mm), grade 3 (perforation 4.1–6.0 mm) and grade 4 (perforation 6.1–8.0 mm). Screws in grade 0, displaced either medially or anteriorly in grade 1 and displaced laterally in grades 1 to 2 were considered acceptable.

**Fig. 2:**
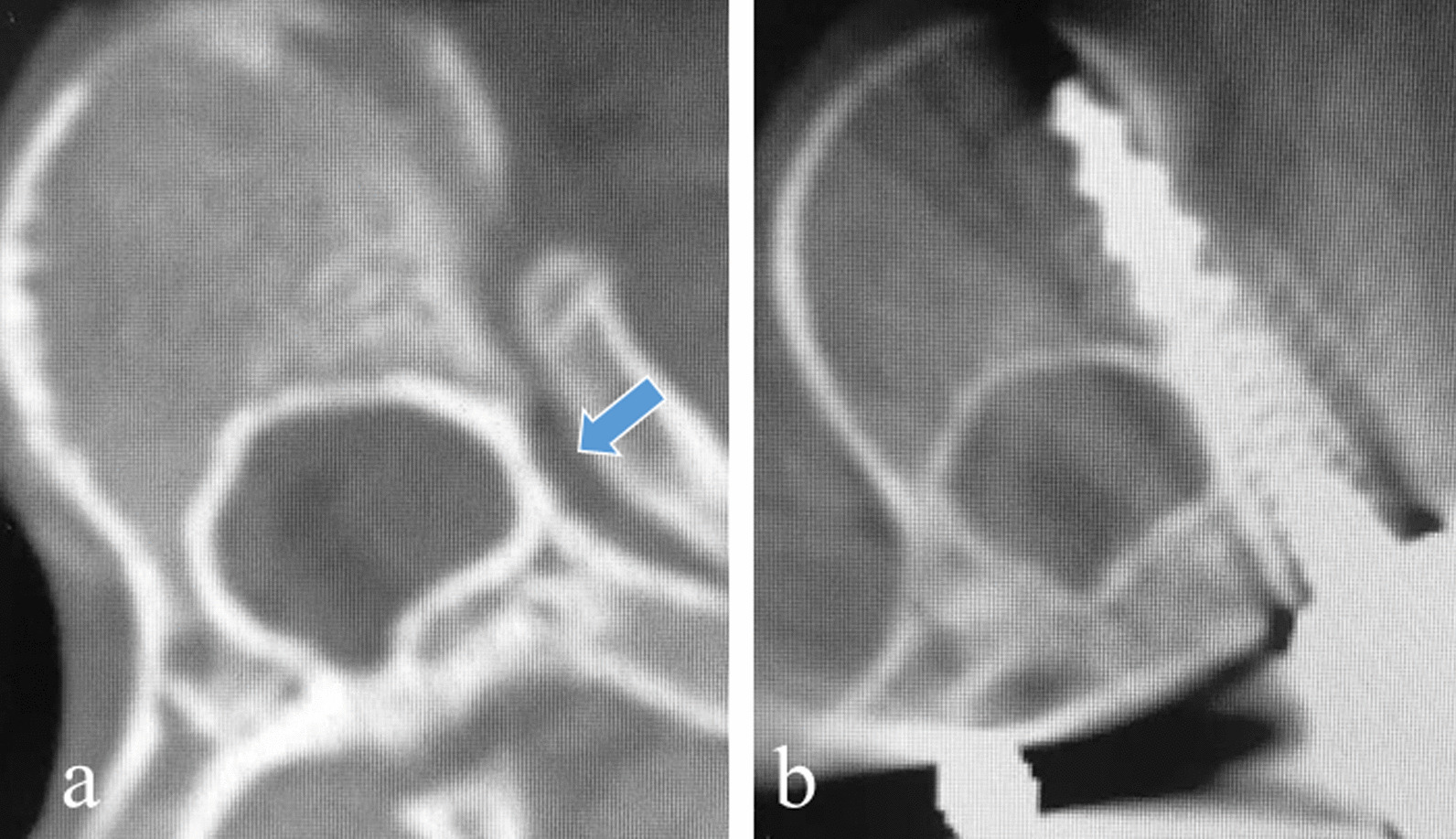
Extrapedicular technique of screw placement in thoracic vertebra with thin pedicle. The width of thoracic pedicle is very small on the concave side (**a** arrow); the concave TP screw passed through the pedicle-rib junction with the screw tip fully contained in the vertebral body (**b**)

Extrapedicular technique of screw placement was used in the thoracic vertebra with extremely small pedicles. For the patients with dysplastic pedicle, the lateral wall of pedicle was intentionally breached and the screw passed through the pedicle-rib junction with the tip of the screw within the vertebral body. Therefore, the screw was fully contained in bony structures and was not considered as laterally displaced although the lateral wall of the dysplastic pedicle was perforated (Fig. 2).

### Statistical analysis

Statistical analysis was performed by SPSS software for Windows (14.0, Chicago, IL). Qualitative variables was analyzed by chi-square test. Significance was established at the P < 0.05 level.

## Results

A total of 495 TP screws were placed in all these cases. The average operation time was 290.1 min and the average blood loss was 870.6 ml. There were 34 screws displaced with the overall perforation rate of only 6.9%. Of these 34 screws, 7 (20.6%) were displaced medially, 20 (58.8%) were displaced laterally and 7 (20.6%) were displaced anteriorly. There were 11 screws (32.4%) in grade 1, 14 screws (41.2%) in grade 2 and 9 screws (26.5%) in grade 3 respectively. All the 9 screws in grade 3 were laterally displaced (Table 1). In addition, we further compared the perforation rate of screws between concave and convex side, and between different thoracic regions. The perforation rate of screws on concave side (6.0%, 17/283) was comparable with that on convex side (8.0%, 17/212) (P > 0.05, Table 2). The perforation rates in the upper (T1–T4), middle (T5–T8) and lower thoracic spine (T9–T12) were 15.7% (13/83), 6.9% (14/202) and 3.3% (7/210), with highest rate in upper thoracic region. (P < 0.05, Table 2). The overall rate of acceptable screws was 97.8%. Neither screw-related complication nor other surgical complication was noted.

**Table 1:** The number and direction of perforation stratified to vertebral level

	Total number	Medial perforation			Lateral perforation			Anterior perforation		
		Grade1	Grade 2	Grade 3	Grade1	Grade 2	Grade 3	Grade1	Grade 2	Grade 3
T1	2	0	0	0	0	0	0	1(convex)	0	0
T2	6	0	0	0	0	0	0	1(concave)	0	0
T3	21	0	0	0	0	0	1(convex)	1(concave)	0	0
T4	54	0	0	0	0	4(1 concave, 3 convex)	1(convex)	3(concave)	1(concave)	0
T5	61	0	0	0	0	0	4(convex)	0	0	0
T6	46	3(2 concave, 1 convex)	1(convex)	0	0	1(convex)	1(convex)	0	0	0
T7	51	1(convex)	0	0	0	0	0	0	0	0
T8	44	1(convex)	0	0	0	2(1 convex, 1 concave)	0	0	0	0
T9	57	0	0	0	0	0	0	0	0	0
T10	44	0	0	0	0	3(concave)	2(concave)	0	0	0
T11	54	0	0	0	1(concave)	0	0	0	0	0
T12	55	0	1(convex)	0	0	0	0	0	0	0

**Table 2:** Comparison of perforation between different sides and different regions

	Perforation	No perforation	*P value
Concave side	17	266	
Convex side	17	195	0.381
Upper thoracic spine (T1–T4)	13	70	
Middle thoracic spine (T5–T8)	14	188	
Lower thoracic spine (T9–T12)	7	203	**0.01**

Boldface indicates statistical significance

*P value calculated using Chi-square analysis

## Discussion

Nowadays, TP screws placement is still a challenging procedure since the maximal permissible translation error is less than 1 mm and the permissible rotation error is less than 5° in thoracic spine[18]. Screw misplacement not only posed a significant threat to adjacent vital neurovascular or visceral structures but also decreased the pull-out strength of the screw, which consequently increased the possibility of instrumentation-related failures [19, 20]. Such risks substantially increased in scoliosis patients with abnormal vertebral morphology and changed anatomic relationship between the vertebrae and surrounding vital structures [21, 22].

To improve the accuracy rate of TP screw insertion in AIS patients, several image-guided techniques had been described, such as fluoroscopy, computer-assisted technique or intraoperative navigation technique, and so on [10–13]. However, these image-guided techniques increased both the radiation exposure and surgery time. The free hand technique was simple and convenient and also exhibit good results of TP screw placements. Since the margin of error for TP screw is small, accurate TP screw insertion with free-hand technique depends on accurate entry point. Many previous studies had described different entry points for TP screws. The Roy-Camille technique selected the intersection between the mid-lines of the facet joint and transverse process as the entry point [23]. Vaccaro reported the entry point to be the junction of the superior border of transverse process and the middle of the superior facet for T4–T9 levels [17]. However, these points had been considered to be inappropriate due to the high incidences of pedicle perforation. Parker identified the entry point in the middle of the triangle formed by the pars interarticularis, the lower edge of the superior articular facet and the medial border of the transverse process. The entry points were more medial and cephalad from T12 to T7 and were more lateral and caudal above T7 [24]. Kim also suggested different entry points at different thoracic regions. The entry point of the 12th thoracic vertebra was located at junction of the bisected transverse process and lamina at the lateral border of the pars. The entry point moved medially and cephalad in the middle thoracic region and moved laterally and caudally in the proximal thoracic region [15]. Although these entry points had been proven to be practicable, they were not quantified, which made the young surgeons without much experience on screw placement confused and increased the risks of pedicle perforations.

To qualitatively identify the ideal entry point for TP screws in AIS patients, Qi used a new anatomic landmark as the points of reference, which was the most concave point at the junction of the transverse process and the folding part of the lamina [25]. Surgeons can select the entry point according to distances between landmark and entry point in both axial plane and sagittal plane suggested by Qi at each level. However, it is not easy for surgeons to remember the different distances from the landmark to entry point at different levels. Modi introduced the concept of an ideal pedicle entry point for TP screw with the freehand technique which is located at the base of the superior facet at the junction of the lateral one third and medial two thirds [18]. However, about 5 mm of inferior articular process overhangs the base of the superior articular process should be removed to expose the entire base of superior facet, which might lead to more bleeding and more operation time.

**Fig. 3:**
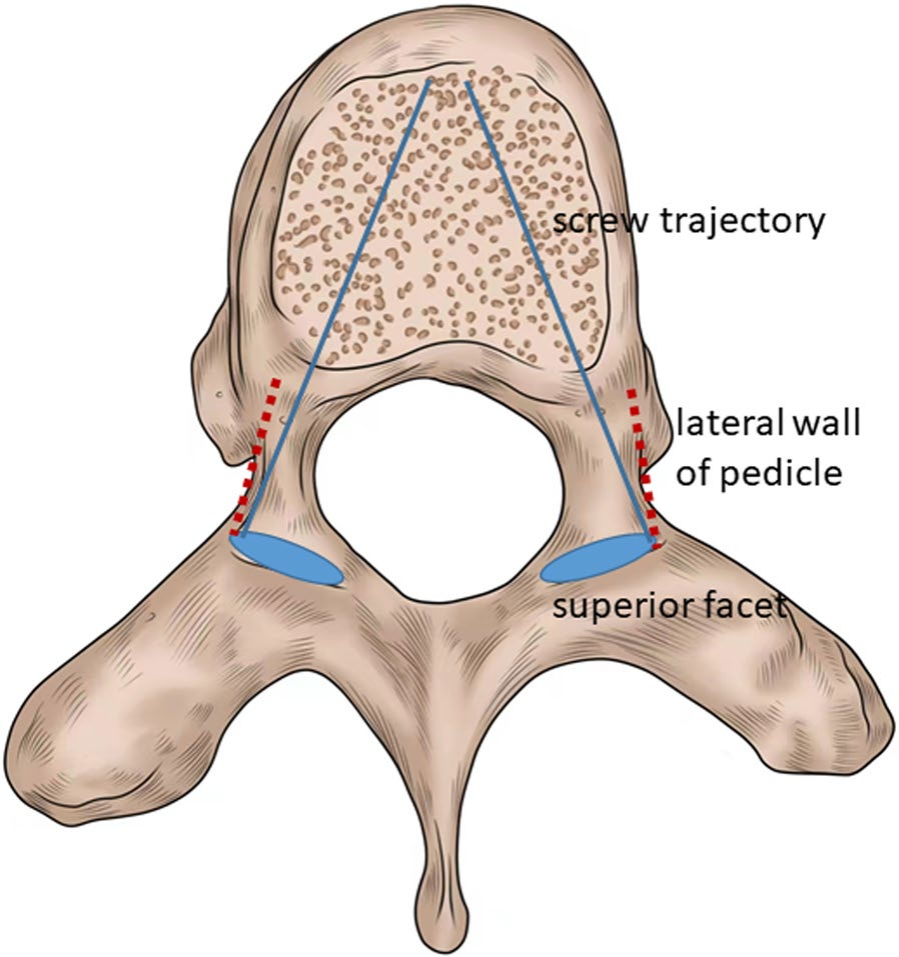
The projection of lateral border of the superior facet on the pedicle was in direct line with lateral wall of the pedicle (red dotted line). Using lateral border of the superior facet as a landmark for identifying screw trajectory is safe

In this study, we propose a constant entry point which can be easily identified without removing the inferior facet. We noticed that in axial plane, projection of lateral border of the superior facet on the pedicle was always located in direct line with lateral wall of the pedicle (Fig. 3). Therefore, the lateral border of the superior facet can be used as a landmark for identifying entry point. Modi selected the entry point at the base of superior facet and no screws showed inferior violation [18]. Therefore, we think the intersection of lateral border of the superior facet and the base of superior facet is a proper entry point for TP screw insertion. However, the lateral edge of the superior facet was often covered by inferior facet, especially on the concave side of thoracic vertebra. Only a curved border connecting lateral border of the superior facet and the transverse process can be seen after exposure of posterior elements. However, removing inferior facet is time-consuming and may increase the blood loss. We thought the intersection of the curved border and inferior facet (Point a in Fig. 1) was approximately the distal end of lateral border of superior facet. O’Brien’s study suggested that the vertebra rotated and shifted as a whole in AIS patients [26]. We presumed that the lateral border of superior facet should be parallel to the supraspinous ligaments at the same level. Therefore, we draw a line from the intersection of the curved border and inferior facet (Point a in Fig. 1), paralleling to the supraspinous ligaments. The intersection of this line and the base of superior facet was selected as the ideal entry point for TP screw insertion in AIS patients. Using this entry point, the overall perforation rate of screw placement was only 6.9% and no screw-related complication happened. Kwan evaluated the accuracy of 2020 pedicle screws from 140 AIS patients and found the total perforation rate was 20.3% with majority of the perforations occurred in thoracic region [9]. In Modi’s study, a total of 152 screws were displaced either medially or laterally in 448 TP screws with the total perforation rate of 33.9% [18]. Qi reported the total perforation rate of 7.8% in 306 TP screws from 21 AIS patients using a landmark determined by CT scan [25]. In the literature, the reported intraoperative blood loss for AIS surgery with pedicle screw instrumentation ranged from 500 to 1212 ml [27–32] and the mean operation time ranged from 146 to 378 min [28, 32–34]. Both the average blood loss and average operation time of our cases were comparable with previous studies. Hence, our method of selecting entry point was both convenient and efficient for TP screw placement in AIS patients.

In the current study, we noticed that all the anterior perforation occurred in the upper thoracic spine, which might explain the highest perforation rate in this region (15.7%). Previous studies had demonstrated that the morphology of proximal thoracic spine was smaller than that of distal thoracic spine and the smaller morphology of proximal thoracic spine was most likely associated with an increased risk of anterior cortical perforation [35, 36]. Jiang’s study reported that the both esophagus and trachea were located in proximity to anterior cortex of proximal thoracic spine in AIS patients [21, 22]. Spine surgeons should choose appropriate screw length to avoid anterior cortex perforation in TP region. As we know, screw placement on the concave side is more difficult than that on the convex side due to the larger converge of the pedicle screw and smaller pedicle diameter. Smorgick reported higher incidence of pedicle perforation on the concave side when compared with the convex side [37]. However, in our study, the perforation rate is similar between the convex and concave side. This result is similar to Min’s study [38]. It is because all the TP screws were placed by the surgeon (J.J) who was experienced on screw placement in AIS patients and was very cautious when inserting screw on the concave side.

There were several limitations which should be mentioned in this study. Firstly, although our new method for selecting the entry point achieved high accuracy of screw placement, the perforation rate in the upper thoracic spine was as high as 15.7%, indicating that this method might be less useful in upper thoracic region. Secondly, the sample size was relatively small. Further study with large sample size is need to verify the accuracy of TPS insertion with our new method. Thirdly, as we know, the accurate TP screw placement depends not only the selection of entry point but also other important steps, such as proper use of awl, the convergence of the screw, and so on. All the TP screws were placed by an attending surgeon (J.J) who was experienced in screw placement. Whether the residents can achieve the same accuracy of screw placement with this method needs further study to evaluate.

## Conclusion

The intersection of the curved border and inferior facet is a reliable landmark for TP screw placement. Using this landmark, high accuracy of screw placement was obtained in AIS patients. We believe that this method is easy to learn for beginners and is worthy of being widely popularized.

## Data Availability

The data and materials in current paper may be made available upon request through sending an e-mail to first author.

## Abbreviations

TP: Thoracic pedicle
AIS: Adolescent idiopathic scoliosis
CT: Computed tomography

## References

[1] Liljenqvist U, Lepsien U, Hackenberg L, Niemeyer T, Halm H (2002). Comparative analysis of pedicle screw and hook instrumentation in posterior correction and fusion of idiopathic thoracic scoliosis. Eur Spine J.

[2] Dobbs MB, Lenke LG, Kim YJ, Kamath G, Peelle MW, Bridwell KH (2006). Selective posterior thoracic fusions for adolescent idiopathic scoliosis: comparison of hooks versus pedicle screws. Spine (Phila Pa 1976).

[3] Kim YJ, Lenke LG, Cho SK, Bridwell KH, Sides B, Blanke K (2004). Comparative analysis of pedicle screw versus hook instrumentation in posterior spinal fusion of adolescent idiopathic scoliosis. Spine (Phila Pa 1976).

[4] Halm H, Niemeyer T, Link T, Liljenqvist U (2000). Segmental pedicle screw instrumentation in idiopathic thoracolumbar and lumbar scoliosis. Eur Spine J.

[5] Di Silvestre M, Parisini P, Lolli F, Bakaloudis G (2007). Complications of thoracic pedicle screws in scoliosis treatment. Spine (Phila Pa 1976).

[6] Kuklo TR, Lenke LG, O’Brien MF, Lehman RA, Polly DW, Schroeder TM (2005). Accuracy and efficacy of thoracic pedicle screws in curves more than 90 degrees. Spine (Phila Pa 1976).

[7] Samdani AF, Ranade A, Sciubba DM, Cahill PJ, Antonacci MD, Clements DH (2010). Accuracy of free-hand placement of thoracic pedicle screws in adolescent idiopathic scoliosis: how much of a difference does surgeon experience make?. Eur Spine J.

[8] Rajasekaran S, Vidyadhara S, Ramesh P, Shetty AP (2007). Randomized clinical study to compare the accuracy of navigated and non-navigated thoracic pedicle screws in deformity correction surgeries. Spine (Phila Pa 1976).

[9] Kwan MK, Chiu CK, Gani SMA, Wei CCY (2017). Accuracy and safety of pedicle screw placement in adolescent idiopathic scoliosis patients: a review of 2020 screws using computed tomography assessment. Spine (Phila Pa 1976).

[10] Viau M, Tarbox BB, Wonglertsiri S, Karaikovic EE, Yingsakmongkol W, Gaines RW (2002). Thoracic pedicle screw instrumentation using the “Funnel Technique”: part 2. Clinical experience. J Spinal Disord Tech.

[11] Kim YW, Lenke LG, Kim YJ, Bridwell KH, Kim YB, Watanabe K (2008). Free-hand pedicle screw placement during revision spinal surgery: analysis of 552 screws. Spine (Phila Pa 1976).

[12] Arand M, Hartwig E, Hebold D, Kinzl L, Gebhard F (2001). Precision analysis of navigation-assisted implanted thoracic and lumbar pedicled screws. A prospective clinical study. Unfallchirurg.

[13] Fu TS, Chen LH, Wong CB, Lai PL, Tsai TT, Niu CC (2004). Computer-assisted fluoroscopic navigation of pedicle screw insertion: an in vivo feasibility study. Acta Orthop Scand.

[14] Cinotti G, Gumina S, Ripani M, Postacchini F (1999). Pedicle instrumentation in the thoracic spine. A morphometric and cadaveric study for placement of screws. Spine (Phila Pa 1976).

[15] Kim YJ, Lenke LG, Bridwell KH, Cho YS, Riew KD (2004). Free hand pedicle screw placement in the thoracic spine: is it safe?. Spine (Phila Pa 1976).

[16] Suk SI, Lee CK, Kim WJ, Chung YJ, Park YB (1995). Segmental pedicle screw fixation in the treatment of thoracic idiopathic scoliosis. Spine (Phila Pa 1976).

[17] Vaccaro AR, Rizzolo SJ, Balderston RA, Allardyce TJ, Garfin SR, Dolinskas C (1995). Placement of pedicle screws in the thoracic spine. Part II: an anatomical and radiographic assessment. J Bone Jt Surg Am.

[18] Modi H, Suh SW, Song HR, Yang JH (2009). Accuracy of thoracic pedicle screw placement in scoliosis using the ideal pedicle entry point during the freehand technique. Int Orthop.

[19] Esses SI, Sachs BL, Dreyzin V (1993). Complications associated with the technique of pedicle screw fixation. A selected survey of ABS members. Spine (Phila Pa 1976).

[20] Faraj AA, Webb JK (1997). Early complications of spinal pedicle screw. Eur Spine J.

[21] Qian B, Jiang J, Zhu F, Zhu Z, Liu Z, Qiu Y (2013). How is the trachea at risk of injury from pedicle screw insertion in proximal thoracic curve of adolescent idiopathic scoliosis patients?. Eur Spine J.

[22] Jiang J, Mao S, Zhao Q, Liu Z, Qian B, Zhu F (2012). Different proximal thoracic curve patterns have different relative positions of esophagus to spine in adolescent idiopathic scoliosis: a computed tomography study. Spine (Phila Pa 1976).

[23] Xu R, Ebraheim NA, Ou Y, Yeasting RA (1998). Anatomic considerations of pedicle screw placement in the thoracic spine. Roy-Camille technique versus open-lamina technique. Spine (Phila Pa 1976).

[24] Parker SL, McGirt MJ, Farber SH, Amin AG, Rick AM, Suk I (2011). Accuracy of free-hand pedicle screws in the thoracic and lumbar spine: analysis of 6816 consecutive screws. Neurosurgery.

[25] Qi DB, Wang JM, Zhang YG, Zheng GQ, Zhang XS, Wang Y (2014). Positioning thoracic pedicle screw entry point using a new landmark: a study based on 3-dimensional computed tomographic scan. Spine (Phila Pa 1976).

[26] O’Brien MF, Lenke LG, Mardjetko S, Lowe TG, Kong Y, Eck K (2000). Pedicle morphology in thoracic adolescent idiopathic scoliosis: is pedicle fixation an anatomically viable technique?. Spine  (Phila Pa 1976).

[27] Crawford AH, Lykissas MG, Gao X, Eismann E, Anadio J (2013). All-pedicle screw versus hybrid instrumentation in adolescent idiopathic scoliosis surgery: a comparative radiographical study with a minimum 2-year follow-up. Spine (Phila Pa 1976).

[28] Carreon LY, Puno RM, Lenke LG, Richards BS, Sucato DJ, Emans JB (2007). Non-neurologic complications following surgery for adolescent idiopathic scoliosis. J Bone Jt Surg Am.

[29] Koerner JD, Patel A, Zhao C, Schoenberg C, Mishra A, Vives MJ (2014). Blood loss during posterior spinal fusion for adolescent idiopathic scoliosis. Spine (Phila Pa 1976).

[30] Diab M, Smith AR, Kuklo TR, Spinal Deformity Study Group (2007). Neural complications in the surgical treatment of adolescent idiopathic scoliosis. Spine (Phila Pa 1976).

[31] Yilmaz G, Borkhuu B, Dhawale AA, Oto M, Littleton AG, Mason DE (2012). Comparative analysis of hook, hybrid, and pedicle screw instrumentation in the posterior treatment of adolescent idiopathic scoliosis. J Pediatr Orthop.

[32] Kwan MK, Loh KW, Chung WH, Chiu CK, Hasan MS, Chan CYW (2021). Perioperative outcome and complications following single-staged Posterior Spinal Fusion (PSF) using pedicle screw instrumentation in Adolescent Idiopathic Scoliosis (AIS): a review of 1057 cases from a single centre. BMC Musculoskelet Disord.

[33] Sugarman E, Sarwahi V, Amaral T, Wollowick A, Gambassi M, Seimon L (2013). Comparative analysis of perioperative differences between hybrid versus pedicle screw instrumentation in adolescent idiopathic scoliosis. J Spinal Disord Tech.

[34] Palmisani M, Dema E, Cervellati S, Palmisani R (2018). Hybrid constructs pedicle screw with apical sublaminar bands versus pedicle screws only for surgical correction of adolescent idiopathic scoliosis. Eur Spine J.

[35] Catan H, Buluç L, Anik Y, Ayyildiz E, Sarlak AY (2007). Pedicle morphology of the thoracic spine in preadolescent idiopathic scoliosis: magnetic resonance supported analysis. Eur Spine J.

[36] Belmont PJ, Klemme WR, Dhawan A, Polly DW (2001). vivo accuracy of thoracic pedicle screws. Spine (Phila Pa 1976).

[37] Smorgick Y, Millgram MA, Anekstein Y, Floman Y, Mirovsky Y (2005). Accuracy and safety of thoracic pedicle screw placement in spinal deformities. J Spinal Disord Tech.

[38] Min WK, Na SB, Jang JA (2018). Accuracy of thoracic pedicle screw placement using freehand technique and triggered EMG in adolescent idiopathic scoliosis: is it different between concave and convex side?. J Orthop Surg (Hong Kong).

